# Supplementary material for: Compensation of the trap-induced quadrupole interaction in trapped Rydberg ions
Source: Sci Rep. 2019 May 14;9:7340. doi: 10.1038/s41598-019-43865-5 (PMC6517410; doi:10.1038/s41598-019-43865-5)
Supplement: Supplementary file 1 — Compensation of the trap-induced quadrupole interaction in trapped Rydberg ions [file 41598_2019_43865_MOESM1_ESM.pdf]

# Supplemental Material for "Compensation of the trap-induced quadrupole interaction in trapped Rydberg ions"

Lachezar S. Simeonov[1], Nikolay V. Vitanov[1] and Peter A. Ivanov[1]
[1] *Department of Physics, St. Kliment Ohridski University of Sofia, James Bourchier 5 blvd, 1164 Sofia, Bulgaria*

## DERIVATION OF THE EFFECTIVE HAMILTONIAN

Following the method presented in Refs. [1, 2] we apply a unitary transformation

$$\begin{aligned}\hat{H}_{\text{eff}} &= e^{i\hat{K}}\hat{H}e^{-i\hat{K}} + i\hbar\left(\frac{\partial e^{i\hat{K}}}{\partial t}\right)e^{-i\hat{K}} = \\ &= \hat{H} + i\left[\hat{K},\hat{H}\right] - \frac{1}{2}\left[\hat{K},\left[\hat{K},\hat{H}\right]\right] - \hbar\frac{\partial\hat{K}}{\partial t} \\ &+ \frac{i\hbar}{2}\left[\frac{\partial\hat{K}}{\partial t},\hat{K}\right] + \frac{\hbar}{6}\left[\hat{K},\left[\hat{K},\frac{\partial\hat{K}}{\partial t}\right]\right] + \ldots.\end{aligned} \tag{1}$$

Our goal is to choose $\hat{K}(t)$ such that $\hat{H}_{\text{eff}}$ becomes time-independent. We expand

$$\begin{aligned}\hat{H}_{\text{eff}} &= \sum_{n=0}^{\infty}\frac{1}{\omega^n}\hat{H}^{(n)}, \\ \hat{K}(t) &= \sum_{n=1}^{\infty}\frac{1}{\omega^n}\hat{K}^{(n)}(t).\end{aligned} \tag{2}$$

The expression $\omega^{-n}\hat{K}^{(n)}$ is $O(\omega^{-n})$, however $\omega^{-n}\partial\hat{K}^{(n)}/\partial t$ is $O(\omega^{-n+1})$.

We start with $0^{\text{th}}$ order. We obtain

$$\hat{H}^{(0)} = \hat{H}_0 + \hat{v}e^{i\omega t} + \hat{v}e^{-i\omega t} - \frac{\hbar}{\omega}\frac{\partial\hat{K}^{(1)}}{\partial t}. \tag{3}$$

We wish to choose $\hat{K}^{(1)}(t)$ such that $\hat{H}^{(0)}$ is time-*in*dependent. To this end

$$\hat{K}^{(1)}(t) = -\frac{i}{\hbar}\left(\hat{v}e^{i\omega t} - \hat{v}e^{-i\omega t}\right). \tag{4}$$

Therefore $\hat{H}^{(0)}$ becomes

$$\hat{H}^{(0)} = \hat{H}_0. \tag{5}$$

We proceed to first order.

$$\begin{aligned}\frac{1}{\omega}\hat{H}^{(1)} &= i\left[\frac{1}{\omega}\hat{K}^{(1)}, \hat{H}^{(0)} + \hat{v}e^{i\omega t} + \hat{v}e^{-i\omega t}\right] \\ &+ \frac{i\hbar}{2}\left[\frac{1}{\omega}\frac{\partial\hat{K}^{(1)}}{\partial t}, \frac{1}{\omega}\hat{K}^{(1)}\right] - \frac{\hbar}{\omega^2}\frac{\partial\hat{K}^{(2)}}{\partial t}.\end{aligned} \tag{6}$$

In order that $\hat{H}^{(1)}$ be time-independent we require

$$\hat{K}^{(2)} = -\frac{2i}{\hbar^2}\left[\hat{v},\hat{H}_0\right]\cos(\omega t). \tag{7}$$

We obtain $\hat{H}^{(1)} = 0$. We continue to second order

$$\frac{1}{\omega^2}\hat{H}^{(2)} = \frac{1}{\hbar^2\omega^2}\left[\left[\hat{v}, \hat{H}_0\right], \hat{v}\right] + \text{time-dependent part} - \frac{\hbar}{\omega^2}\frac{\partial \hat{K}^{(3)}}{\partial t}. \tag{8}$$

We choose $\hat{K}^{(3)}(t)$ to cancel the time-dependent part. Thus we obtain

$$\hat{H}_{\text{eff}} = \hat{H}_0 + \frac{1}{(\hbar\omega)^2}\left[\left[\hat{v}, \hat{H}_0\right], \hat{v}\right]. \tag{9}$$

After lengthy calculation one can show that $\hat{H}^{(3)} = 0$. The next order of magnitude is the fourth order. This may explain the very good agreement between the full Hamiltonian and the effective Hamiltonian even when the Rabi frequencies $\Omega_i$ are increased as high as $\sim \omega/4$.

## DIPOLE-DIPOLE INTERACTION BETWEEN MICROWAVE-DRESSED RYDBERG IONS

We examine two ions, which are microwave dressed. This means that each of the ions is a four-level system as in Fig. 1. However we assume that level $|1\rangle$ is a *Rydberg* state and the detuning $\Delta_2$ is much greater than all other Rabi frequencies and detuning. In this way, level $|1\rangle$ can be adiabatically eliminated. Following [3] we have

$$\hat{H} = \sum_{j=1}^{2}\{\Delta_2'|2_j\rangle\langle 2_j| + \Delta_3|3_j\rangle\langle 3_j| + \Omega_2\left(|2_j\rangle\langle 3_j| + |3_j\rangle\langle 2_j|\right)\} + \sum_{j=1}^{2}\Omega\cos(\omega t)\left(|2_j\rangle\langle 4_j| + |4_j\rangle\langle 2_j|\right) + \hat{H}_{\text{dd}}, \tag{10}$$

where $\Delta_2' = \Omega_1^2/\Delta_1$ is a Stark shift of level $|2\rangle$ and $\hat{H}_{\text{dd}}$ is the dipole-dipole interaction

$$\hat{H}_{\text{dd}} = \lambda\left(|2_1 3_2\rangle\langle 3_1 2_2| + |3_1 2_2\rangle\langle 2_1 3_2|\right) + \mu|2_1 2_2\rangle\langle 2_1 2_2|, \tag{11}$$

where

$$\mu = \frac{2\Omega_1^2}{\Delta_1^2}\frac{|\langle 2|\hat{d}_x|3\rangle|^2 + |\langle 2|\hat{d}_y|3\rangle|^2 - 2|\langle 2|\hat{d}_z|3\rangle|^2}{8\pi\epsilon_0|z_0^{(1)} - z_0^{(2)}|^3}. \tag{12}$$

The effective Hamiltonian becomes

$$\begin{aligned}\hat{H}_{\text{eff}} = &\sum_{j=1}^{2}\left(\Delta_2'\left(1 - \frac{\Omega^2}{2\omega^2}\right)|2_j\rangle\langle 2_j| + \Delta_3|3_j\rangle\langle 3_j| + \Delta_2'\frac{\Omega^2}{2\omega^2}|4_j\rangle\langle 4_j| + \Omega_2\left(1 - \frac{\Omega^2}{4\omega^2}\right)\left(|2_j\rangle\langle 3_j| + |3_j\rangle\langle 2_j|\right)\right) \\ &+ \lambda\left(1 - \frac{\Omega^2}{2\omega^2}\right)\left(|2_1 3_2\rangle\langle 3_1 2_2| + |3_1 2_2\rangle\langle 2_1 3_2|\right) + \mu\left(1 - \frac{\Omega^2}{\omega^2}\right)|2_1 2_2\rangle\langle 2_1 2_2| \\ &+ \lambda\frac{\Omega^2}{2\omega^2}\left(|4_1 3_2\rangle\langle 3_1 4_2| + |3_1 4_2\rangle\langle 4_1 3_2|\right) + \mu\frac{\Omega^2}{2\omega^2}\left(|4_1 2_2\rangle\langle 4_1 2_2| + |2_1 4_2\rangle\langle 2_1 4_2|\right) \\ &+ \mu\frac{\Omega^2}{2\omega^2}\left(|2_1 4_2\rangle\langle 4_1 2_2| + |4_1 2_2\rangle\langle 2_1 4_2|\right) - \mu\frac{\Omega^2}{2\omega^2}\left(|2_1 2_2\rangle\langle 4_1 4_2| + |4_1 4_2\rangle\langle 2_1 2_2|\right).\end{aligned} \tag{13}$$


[1] Goldman, N. & Dalibard, J. Periodically driven quantum systems: effective Hamiltonians and engineered gauge fields. *Phys. Rev. X*, **4**, 031027 (2014).

[2] Rahav, S., Gilary, I. & Fishman, S. Effective Hamiltonians for periodically driven systems. *Phys. Rev. A* **68**, 013820 (2003).

[3] Müller, M., Liang, L., Lesanovski, I. & Zoller, P. Trapped Rydberg ions: from spin chains to fast quantum gates. *New. J. Phys.* **10**, 093009 (2008).
